# Creating information resources and trainings for farmworker-serving community health workers

**DOI:** 10.5195/jmla.2022.1272

**Published:** 2022-01-01

**Authors:** Jamie E. Bloss, Catherine E. LePrevost, Leslie E. Cofie, Joseph G. L. Lee

**Affiliations:** 1 blossj19@ecu.edu, Assistant Professor, Laupus Health Sciences Library, East Carolina University, Greenville, NC; 2 celeprev@ncsu.edu, Associate Extension Professor, Department of Applied Ecology, NC State University, Raleigh, NC, and NC Agromedicine Institute, Greenville, NC; 3 cofiel18@ecu.edu, Assistant Professor, Department of Health Education and Promotion, College of Health and Human Performance, East Carolina University, Greenville, NC; 4 leejose14@ecu.edu, Associate Professor, Department of Health Education and Promotion, College of Health and Human Performance and Center for Health Disparities, Brody School of Medicine, East Carolina University, Greenville, NC

**Keywords:** farmworkers, health literacy, community health workers, health promotion, information literacy, healthcare disparities, minority health

## Abstract

**Background:**

Farmworker-serving community health workers have limited access to farmworker health research findings, training, and education resources. With funding from the National Library of Medicine, we are working to improve the health information literacy of both community health workers and farmworkers. We conducted focus group discussions with community health workers to explore their experiences providing health education and information to farmworkers, their information-seeking behaviors, and their technology and information needs. Data from the focus groups provided insights into the main areas in which community health workers would like to receive professional development.

**Case Presentation:**

Our team, which includes health sciences librarians, developed a resource list of educational materials for farmworker health, videos to increase community health workers' skills finding health information online, and webinars to introduce these resources to community health workers. Videos, available in Spanish and English, included instruction on finding and evaluating online health information, accessing reputable online consumer health information sources, and advanced searching tips for Google and PubMed. Through three webinars, we introduced the resource list, videos, and design software for creating handouts and infographics to community health workers.

**Conclusions:**

Community health workers have a critical role in providing health education and information to farmworkers, and our efforts represent a first step in addressing community health workers' limited access to professional development. Health sciences librarians are well positioned to partner with interdisciplinary teams working to reduce health disparities and provide resources and training to community health workers, farmworkers, and other underserved communities.

## BACKGROUND

Migrant and seasonal farmworkers face significant health disparities [1]. Migrant and seasonal farmworkers are “individuals] whose principal employment is in agriculture on a seasonal basis,” and migrant farmworkers have to establish a temporary abode for the purposes of such employment [2]. Farmworkers in the US tend to be foreign-born (75%) and Spanish speaking (77%); the majority of them come from Mexico or Central American countries [3,4]. Farmworkers are at greater risk for many noncommunicable diseases, mental health issues, heat stress, pesticide exposure, and other occupational hazards [1]. They also often lack access to health and safety information [5]. Many farmworkers are undocumented and live below federal poverty levels, which diminishes their access to legal protections and health care [4].

In order to address farmworker health disparities, with funding from the National Library of Medicine (NLM) (Grant #G08LM013198), we are working to improve the health information literacy of bothfarmworkers and the community health workers (CHWs) who provide farmworkers with health education and information. Our team includes two public health faculty from East Carolina University, an agromedicine faculty member from NC State University, and the executive director from Student Action with Farmworkers. Two health sciences librarians on the team, from Laupus Health Sciences Library, provide library support and NLM product expertise for the project. Our project aims to improve the capacity of CHWs to provide health education and related resources to farmworkers, and to provide farmworkers with adequate access to health information resources and Internet access.

A CHW is a frontline public health worker who is a trusted member and can have an unusually close understanding of the community they serve [6]. Farmworker-serving CHWs in North Carolina (NC) tend to be female, Spanish-speakers, and Latinx [7]. Whereas CHWs serve an important role in providing health care access and enabling services (e.g., health education, interpretation, and case management) for farmworkers, CHWs have received limited attention in the literature [8]. Furthermore, our previous research involving farmworker-serving CHWs has demonstrated they have limited access to farmworker health research findings and other important health information resources [9]. Our current project strives to address this gap through training that increases CHWs' health information literacy skills and their knowledge of NLM resources. To inform this training, we conducted three focus group discussions (FGDs) with CHWs in NC to explore their experiences providing health education and information, their information-seeking behaviors, and their technology and information needs. Data from these FGDs provided insights into the main areas in which CHWs would like to receive professional development. We developed training materials (e.g., videos, resource list) and associated webinars for the CHWs based on these insights.

Analysis of the focus group transcripts revealed that priority areas for CHW professional development included finding and evaluating online health information, accessing reputable online consumer health information sources, and advanced searching tips for Google and PubMed. A prevailing concern described by CHWs was repeatedly presenting the same materials to farmworkers season after season. CHWs perceived this repetition as ineffective and mentioned the constant need for new content. When describing searching for health information online, one CHW explained, “We want to make sure that we're getting it based [in research]—we don't look at every ad that comes up on the computer because sometimes it can be false information.” Several CHWs mentioned searching for evidence-based information from reputable sources such as the Centers for Disease Control and Prevention. Some CHWs also said that they reformulate health information that is too advanced, not farmworker specific, or written in English into health information that is culturally, linguistically, and educationally appropriate for a farmworker audience.

Focus group findings indicated that many CHWs perceive themselves as capable of searching online for reliable health information. We know that individuals tend to overestimate their online searching abilities [10], and as a result, we wanted to also provide a basic introduction to searching and evaluating information online. In response to the focus group findings, we created a resource list of existing educational materials, a series of videos, and webinars for CHWs to introduce them to these resources as well as to design software to make handouts and infographics.

## CASE PRESENTATION

### Creation of resource list of educational materials for farmworker health

We created a resource list of educational materials for farmworker health, which was shared with CHWs. To do this, we first identified and assessed health information resources designed for farmworkers and identified gaps in available resources. We identified sources of materials by asking our advisory panel members, researchers we knew in the field, outreach organizations, and existing partners, and by running Google searches. We then acquired materials from various farmworker health organizations' websites and solicited materials from these organizations by mail and email. We compiled a listing of the consumer health information pamphlets, handouts, flipcharts, *fotonovelas,* and any other materials that could be used with farmworkers during outreach. A Patient Education Materials Assessment Tool (PEMAT) assessment was conducted on the materials by our team [11]. These materials were organized in a searchable document by audience, which included publications aimed at health educators first, then materials for a farmworker audience, followed by generic agricultural resources, and materials for farm owners. Within each of those sections, materials were further organized by topic. Topics included information on alcohol and drug usage, arthritis and musculoskeletal issues, clinic services, cancer, diabetes, emotional or mental health, first aid, green tobacco sickness, hand washing and sanitation, heat and sun safety, sexually transmitted infections, insurance, pesticides, safety and injury prevention, sexual harassment and intimate partner violence, and much more. Information on the language, publication type, and title of the material was included with each link. Because we do not hold the copyrights for the materials, we linked all materials back to the original websites. The document included 547 links and was placed on the UNC Dataverse and linked to our project website [12]. The downloadable document can be found in the UNC Dataverse [13].

### Creation of videos

We created four videos for CHWs. First, we created a video on searching for online information and evaluating information using the Currency, Relevance, Authority, Accuracy, and Purpose (CRAAP) test. A second video introduced free consumer health resources, including MedlinePlus.gov, healthfinder.gov, health.gov, mayoclinic.org, health.harvard.edu, and thecommunityguide.org. We also included healthreach.gov at the time, which has since been discontinued. We also created two more advanced videos—one on the Google Advanced search function to narrow down content on Google or filter by domain and one on running a basic search in PubMed.gov. For each video, we made sure to include only resources that were freely accessible as CHWs may not have library access for conducting literature searches or using databases. In the PubMed.gov video, we showed how to access any free articles and how to use Google Scholar or researchgate.net to access full-text versions of articles. Videos were approximately ten to fifteen minutes in duration each [14].

Both English- and Spanish-language versions of the four video topics were made available in order for participants to choose their preferred language. Each video script was written in English and translated into Spanish by a professional translator. We used Camtasia screen recorder software for the videos so we could demonstrate searching and accessing the various websites. The health sciences librarian recorded the four English language versions of the videos, and a Physician Assistant Studies student whose first language is Spanish recorded the four Spanish language versions. We included subtitles in the recorded language for each video.

### Dissemination via webinars

Over the course of three webinars, we introduced to CHWs the resource list, videos, and design software to make handouts and infographics. We had initially planned to provide regional, in-person professional development for CHWs, but we transitioned to statewide, online webinars as a result of the COVID-19 pandemic. All webinars were advertised through direct mailings to CHWs who had participated in FGDs as well as through emails forwarded via the listservs of our community partners, including the NC Farmworker Health Program and the NC Community Health Center Association. We offered the webinars in either Spanish (first webinar) or English (second and third webinars) with simultaneous interpretation provided in the other language.

#### First webinar

The first webinar, New Technology and Resources for Outreach, was held on May 27, 2020, for thirty-two participants. During this sixty-minute webinar, we reported back key findings from the FGDs and introduced the resource list. We led participants through several interactive activities during which scenarios were presented where CHWs might need to access farmworker educational materials, and participants practiced using the resource list to find relevant materials. Scenarios included finding resources for a farmworker woman requesting information about a recent diagnosis of type 2 diabetes, materials for a CHW wanting to learn more about domestic violence after suspecting a case of abuse, and a curriculum for an ergonomics workshop for farmworkers.

#### Second webinar

The second webinar was held in conjunction with a regularly scheduled “coffee corner” hosted by our community partner, the NC Farmworker Health Program. The thirty-minute webinar was held on November 6, 2020. During this webinar, Finding Up-to-Date and Trustworthy Health Information Online, we introduced the videos to twelve participants. Also, we provided a virtual tour of the project website (https://www.farmworkerhealthliteracy.org/for-outreach-workers), showing participants where to access both the videos and the resource list that was introduced during the first webinar. At the end of the second webinar, we asked participants to prepare for the third webinar by selecting a health topic they would like to share with farmworkers and, using what they learned from watching the videos, come up with two or three important take-home messages they would want farmworkers to understand about that topic. We did not present these webinars as a series and had some different participants at each event. We did advertise and disseminate the information to the same groups of people each time in hopes they could come to the follow-up webinars.

#### Third webinar

**Figure 1:**
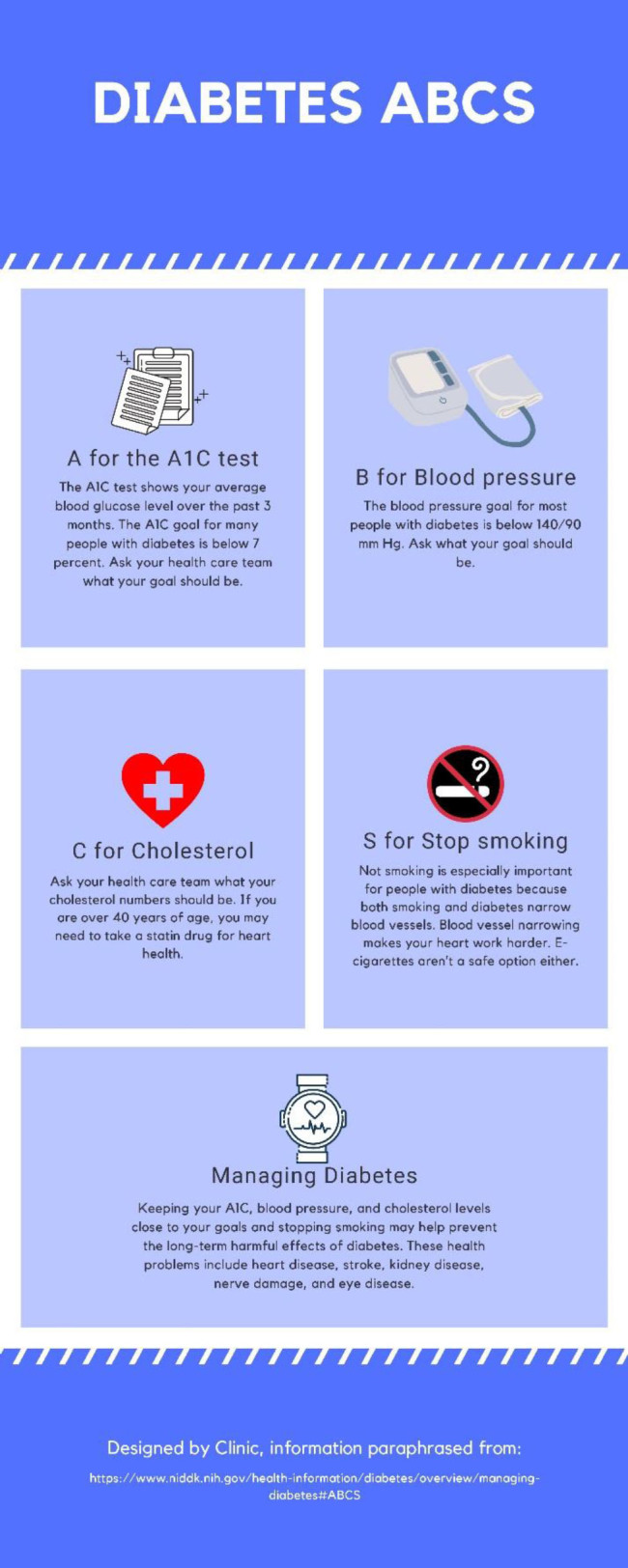
The infographic we created as an example of a design during the third webinar. This was not intended for use with farmworkers but as an example of the layout and design functions within Canva.com.

The third webinar, Finding Trustworthy Information Online and Creating Infographics for Farmworkers, was a sixty-minute session held on November 18, 2020, with seventeen participants. During this webinar, which was hosted by the health sciences librarian, we included a question-and-answer session on the videos, a presentation on tips for how to present consumer health information and create more effective patient education materials, and a step-by-step tutorial on how to use Canva.com to create an infographic for dissemination online via WhatsApp or social media. Canva.com is an online design website, which offers some free features. We utilized these free features during the webinar. Additionally, we showed options for making a poster or flyer in Canva.com and resources available through Canva Design School and tutorial videos. During the webinar, we created an example infographic to show the CHWs how to create a design in Canva.com (Figure 1). For this example and throughout the webinar, we focused on high blood pressure and diabetes management, common farmworker health concerns.

### Evaluation of CHW resources

We are conducting a utilization-focused process evaluation of the overall grant project, including the resources and webinar preparation and implementation. The purpose of this evaluation is to provide usable information to our team and partners for program and material improvements. The evaluation is driven by a logic model and includes interviews with CHWs and farmworkers and tracking of metrics on material use. The diverse expertise of our research team—including public health, library science, science education, and farmworker advocacy—helped inform the resource and webinar development. Additionally, we received preliminary feedback via email from our project's advisory board members, our community partners, and participating CHWs. One advisory board member remarked on the videos, “Wow … this is really well-done and such a great tool for [community health] outreach workers. It is great that the videos are under 20 minutes!” A community partner likewise praised the videos and expressed her appreciation for the opportunity to review draft scripts of the videos: “This video is a great tool that can be extremely beneficial for those new to public health, outreach, and community health. Thanks again for the opportunity to share our thoughts in the development process.” Remarking on the resource list, one CHW participating in the webinars said, “Thank you for this information … this will work a lot for me this year, and years to come.” Another CHW said of project materials, “I think [this project] is going the right way to share information with the farmworkers … All good ideas that I can't wait to use all the resources. Job well done!!” We plan to conduct a survey evaluation of CHWs' experiences and benefits derived from the resources after more have had the opportunity to implement lessons learned into their farmworker outreach efforts.

## DISCUSSION

Informed by FGDs with CHWs about their experiences conducting outreach to farmworkers and their technology and information needs, we developed a resource list of educational materials for farmworker health, videos to increase CHWs' skills finding health information online, and webinars to introduce these resources to CHWs. Given the critical importance of CHWs in farmworker health, this represents only the first step in addressing the limited access to professional development for these health professionals. It is vitally important that the information and training needs of CHWs continue to be addressed. In addition to the paucity of resources for CHWs, there is little consumer health information on the web specifically tailored for farmworkers. Our project addressed this gap by collaborating with CHWs to facilitate opportunities for them to learn how to create their own instructional materials for their outreach.

Originally, we had planned to offer regional, in-person workshops to introduce resources and provide training to CHWs. Because of the COVID-19 pandemic and resulting travel and gathering restrictions, we transitioned to providing statewide, online webinars. This certainly widened the reach of whom we could meet with across the state at one time, but due to broadband issues, this could also have been a barrier to accessing the session for participants in some parts of the state whom we might have reached out to in person. Another challenge we experienced was uncertainty as to how the pandemic would impact the seasonal nature of CHWs' schedules [3]. During the agricultural season, CHWs spend a significant portion of their time providing health education, health assessments, and other services to farmworkers. The offseason, in which they are not working with farmworkers every day, is typically a time of intense preparation for future outreach and finding additional resources for farmworkers. During the pandemic, CHWs were further stretched to provide digital outreach (e.g., distributing health information via WhatsApp and scheduling telemedicine appointments), facilitate COVID-19 testing, respond to COVID-19 outbreaks in farmworker housing, and plan for vaccine distribution. We observed better participation in the webinar we offered earlier in the pandemic than in the later ones. We speculate that attendance was lower for the November webinars (which came at the end of the agricultural season) due to CHW fatigue—both from the challenging season and an increased reliance on the web to conduct most work-related functions. Another challenge was that any time you are creating educational resources, they need to be updated and maintained. For example, with the healthreach.gov database being discontinued, already one of our videos is out of date and will need to be edited and re-uploaded online. This is certainly the case with the large PDF resource list of educational materials we created as well, as links may change, and link rot is a pervasive Internet issue.

The literature on migrant and seasonal farmworker health has limited information on the resources available to CHWs in their work to provide critical health education and information to farmworkers [2,8]. This dearth may be due, in part, to the lack of a formalized position for CHWs, which would acknowledge the complex responsibilities CHWs assume in providing both social and health-enabling services to farmworkers. As efforts are underway to formalize the CHW position (e.g., The Community Health Worker Core Consensus (C3) Project: c3project.org), we anticipate that the needs and opportunities to provide professional development for CHWs will only expand and that librarians will have a critical role in this initiative.

Because many librarians have a background in education and experience working with many levels of information-literate patrons, our team, which includes librarians, is uniquely poised to work with diverse groups of people and teach skillsets that go beyond searching, including creating effective instructional materials. Medical and health sciences librarians can support the information and training needs of similar projects and partnerships with schools of public health, farmworker health organizations, advocacy groups, and more. Health sciences librarians can work to reduce health disparities and provide consumer health information searching instruction to CHWs, to farmworkers directly, and to other underserved communities. To gain better insight into the communities CHWs serve and develop relevant and appropriate materials, we suggest opportunities for health sciences librarians to work alongside CHWs in the future. Our librarian and project partners plan to continue offering training and to support this collaborative work in the future.

## Data Availability

All datasets associated with this project are available at: https://dataverse.unc.edu/dataverse/farmworkerhealth.
