# Erratum to: Bloss JE, LePrevost CE, Cofie LE, Lee JGL. Creating information resources and trainings for farmworker-serving community health workers. J Med Libr Assoc. 2022;110(1):113–118. DOI: https://doi.org/10.5195/jmla.2022.1272.

**DOI:** 10.5195/jmla.2022.1497

**Published:** 2022-01-01

**Authors:** Jamie E. Bloss

The following funding disclosure was left out of the manuscript during the submission and production process. The original manuscript has been updated to include this statement.

## FUNDING STATEMENT

Research reported in this publication was supported by the National Library of Medicine of the National Institutes of Health under Award Number G08LM013198. The content is solely the responsibility of the authors and does not necessarily represent the official views of the National Institutes of Health.

